# Evaluation of screening methods for preclinical interstitial lung disease associated with rheumatoid arthritis

**DOI:** 10.36416/1806-3756/e20240402

**Published:** 2025-09-22

**Authors:** Ana Luisa Bagno de Almeida, Mariana Drummond Martins Lima, Maria Fernanda B. Resende Guimarães, Eliane Viana Mancuzo

**Affiliations:** 1. Universidade Federal de Minas Gerais, Belo Horizonte (MG), Brasil.

## TO THE EDITOR,

The early diagnosis of rheumatoid arthritis-associated interstitial lung disease (RA-ILD) remains a challenge in clinical practice, and the development of individualized screening criteria using measurable indicators is still an unmet need that warrants further investigation.^1^ The most recent guidance on the subject-the 2023 American College of Rheumatology (ACR)/American College of Chest Physicians (CHEST) Guideline for the Screening and Monitoring of Interstitial Lung Disease (ILD) in People with Systemic Autoimmune Rheumatic Diseases-recommends screening all patients considered at high risk for RA-ILD using high-resolution computed tomography (HRCT) and spirometry with lung volumes and diffusing capacity of carbon monoxide (DLCO). A conditional recommendation against the use of the six-minute walk test (6MWT) was issued due to the lack of studies evaluating its efficacy as a screening tool.^2^

In order to evaluate the association between the 6MWT, serum levels of Krebs von den Lungen-6 (KL-6), and spirometry parameters, including lung volumes and DLCO, with HRCT findings suggestive of ILD in RA, we conducted a cross-sectional observational study at a tertiary rheumatology center in Brazil. All included participants were adults, with a confirmed diagnosis of RA according to the 2010 ACR/EULAR criteria,^3^ without respiratory symptoms or a prior diagnosis of ILD, and had been on stable treatment for at least 8 weeks. All RA diagnoses were reviewed and confirmed, and data were collected through clinical evaluations and medical record reviews. 

The study participants underwent HRCT to identify ILD findings, including the presence of reticulation with or without ground-glass opacities, traction bronchiectasis, honeycombing, or isolated ground-glass opacities. Two independent, qualified readers, who were not blinded to the clinical data, evaluated the HRCT scans. Serum KL-6 levels were measured using the ELISA technique. Pulmonary function tests (PFTs), including the 6MWT, were performed according to international guidelines.^4^ ^,^ ^5^ Although all assessments were intended to be conducted on the same day, eleven patients had an interval of more than 100 days between tests due to team or patient unavailability. Nevertheless, this was deemed acceptable, as no significant clinical events occurred during this period. Overall, the time intervals between tests were short: the median interval between HRCT and PFTs was 20 days; between KL-6 and PFTs, 35 days; and between HRCT and KL-6, 0 days. Association and concurrent validity analyses were performed using Pearson’s correlation coefficient, biserial correlation, and point-biserial correlation tests. Receiver operating characteristic (ROC) curves were constructed to determine cutoff values. Data were analyzed using SPSS (version 23.0), JASP (version 0.18.3.0), and Jamovi (version 2.5.3). 

This study was approved by the local ethics committee, and all patients provided informed consent.

Initially, a non-probability sample of 44 patients was selected. However, 7 were unable to complete all the required assessments, resulting in a final cohort of 37 patients. The majority were female (86.5%), and 81% had elevated levels of rheumatoid factor and/or anti-cyclic citrullinated peptide (anti-CCP) antibodies. The mean age of the patients was 54.4 years (SD=13.75), with a median disease duration of 4.6 years. Additionally, 37.8% were current or former smokers (Table 1). 

**Table 1:** Clinical characteristics of the study sample (n=37).

Variables	Descriptive Statistics
Age, years	54.41 (13.75)
Female sex, n / %	32 / 86.5
Smoking, n / % Ex-smoker Current smoker Never smoked Tobacco load, pack-years	12 / 32.4 2 / 5.4 23 / 62.2 18.87 (30.98)
Disease duration, years*	4.60 (0.56-50.56)
Rheumatoid factor, n / % Low: <3x ULN High: >3x ULN No information	7 / 18.9 22 / 59.5 8 / 21.6
Anti-CCP, n / % Low: <3x ULN High: >3x ULN No information	2 / 5.4 27 / 73.0 8 / 21.6
DAS-28 CRP	3.46 (1.33)
CDAI	13.20 (11.96)
HAQ	1.17 (0.96)
CRP	10.4 (8.59)
Charlson Comorbidity index	2.54 (1.57)
MEAS, n / %	2 / 5.4
Current medications, n / % Corticosteroids Anti-TNF Rituximab Tocilizumab iJAK	20 / 54.1 3 / 8.1 3 / 8.1 1 / 3.7 4 / 10.8
Synthetic DMARDs, n / % MTX HQC LFN SSZ AZA	22 / 59.5 1 / 2.7 18 / 48.6 0 / 0.0 1 / 2.7

ULN: upper limit of normal; Anti-CCP: anti-cyclic citrullinated peptide antibody; DAS-28 CRP: Disease Activity Score-28 with C-Reactive Protein; CDAI: Clinical Disease Activity Index for Rheumatoid Arthritis; HAQ: Health Assessment Questionnaire; CRP: C-Reactive Protein; MEAS: extra-articular manifestations; Anti-TNF: tumor necrosis factor antagonist; iJAK inhibitor: Janus kinase inhibitor; DMARD: disease-modifying antirheumatic drug; MTX: methotrexate; HCQ: hydroxychloroquine; LFN: leflunomide; SSZ: sulfasalazine; AZA: azathioprine. *Data expressed as mean (standard deviation), absolute/relative frequency, and median (minimum and maximum values).

The HRCT scans revealed mild findings, affecting less than 5% of the lung parenchymal volume according to the semi-quantitative evaluation by the readers, with no evidence of honeycombing. Only 29.8% of the scans were normal. Interstitial changes were observed in 32.4% of the scans, with one case (2.7%) showing overlap with airway alterations. Non-specific and airway-related changes-such as nodules, cysts, emphysema, and air trapping-were identified in 35.1% of the scans. 

On average, the patients exhibited a forced vital capacity (FVC) of 1.96 ± 0.69 L (88.8 ± 18.77% of predicted), a forced expiratory volume in the first second (FEV_1_) of 2.38 ± 0.59 L (89.92 ± 12.54% of predicted), and an FEV_1_/FVC ratio of 80.0 ± 7.64-all values above the lower limit of normal. Two tests indicated a mild restrictive pattern, six showed a mild-to-moderate obstructive pattern, and one revealed a combined ventilatory defect. The mean DLCO was 19.02 ± 4.0 mL/min/mmHg (96.53 ± 16.18% of predicted), with 10.8% of patients showing a mild reduction. The mean serum KL-6 level was 606 ± 362.64 U/mL.

**Figure 1:**
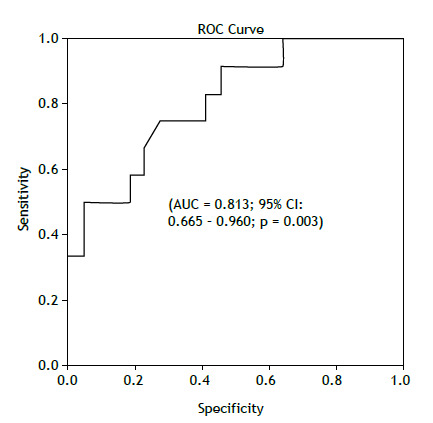
ROC curve from distance of 6MWT (cutoff = 462.55 m).

The mean 6MWT distance (6MWTD) was 451.08 ± 105.58 meters and was moderately correlated with preclinical ILD changes on HRCT (r_p_b=0.547; p=0.001). In our study, a 6MWTD cutoff of 462.55 m demonstrated good accuracy in distinguishing patients with HRCT findings suggestive of RA-ILD from those without (AUC=0.813; 95% CI: 0.665 - 0.960; p=0.003) (Figure 1). Serum KL-6 levels were also correlated with HRCT findings suggestive of ILD (r_p_b=0.33; p=0.044) and showed a trend toward differentiating early interstitial changes from normal scans in this population (p=0.043). However, KL-6 levels did not distinguish ILD from other abnormalities (p=0.593), and the ROC curve did not reach statistical significance (p=0.61). Regarding spirometry, absolute DLCO values (r_p_b=0.46; p=0.006), FVC (r_p_b=0.43; p=0.008), and FEV_1_ (r_p_b=0.42; p=0.010) were also associated with RA-ILD findings on HRCT. 

The findings related to the 6MWT in this study were promising. While the prognostic value of the 6MWT in ILD is well established,^6^ its potential role as a screening tool for preclinical ILD remains largely unexplored. In an exploratory analysis of patients with RA-ILD (n=54), Boudal et al. (2024) reported a mean 6MWTD of 267.7 m; however, their cohort included patients with respiratory symptoms (30.2% with dyspnea and 55.6% with cough).^7^

Regarding KL-6, elevated serum levels have been associated with the presence and severity of RA-ILD,^8^ although evidence supporting its utility in detecting preclinical disease is still limited. Similarly, previous studies have linked reduced FVC and DLCO values with increased disease severity in RA-ILD,^9^ as well as with the presence of interstitial lung abnormalities in otherwise healthy individuals.^10^ Given that RA-ILD symptoms often emerge late in the course of the disease,^1^ early, non-invasive screening tools such as the 6MWT or KL-6 measurement could help identify patients who may benefit from further evaluation with HRCT.

This study assessed the association between screening test results and the diagnosis of preclinical RA-ILD, as identified on HRCT. These findings should be interpreted with caution due to the small sample size, which may limit the robustness and generalizability of the conclusions. Validation in larger cohorts is necessary to confirm these results and to further investigate their potential role in refining RA-ILD screening strategies. Nonetheless, in this cohort of asymptomatic RA patients with high autoantibody titers, a 6MWD of less than 462.55 meters was significantly associated with the presence of interstitial abnormalities on HRCT. Elevated serum KL-6 levels also demonstrated an association, reinforcing the potential value of these biomarkers in RA-ILD risk assessment.
